# Sensory limits in the rodent whisker system predict an internal forward model for sensorimotor estimation of object touch

**DOI:** 10.1186/1471-2202-12-S1-P101

**Published:** 2011-07-18

**Authors:** Peter Gyring, A Aldo Faisal

**Affiliations:** 1 Department of Bioengineering, Imperial College London, London, SW7 2AZ, UK; 2 Department of Computing, Imperial College London, London, SW7 2AZ, UK

## 

**Figure 1:**
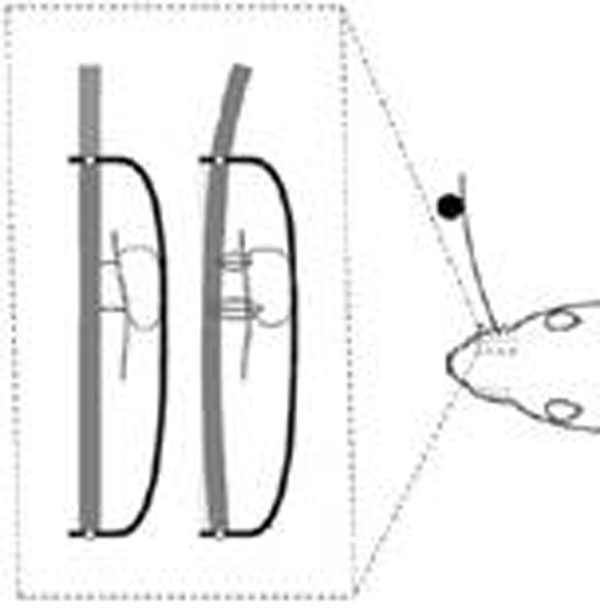
Whisker bending within follicle upon object contact

The rodent whisker system is an important model system for active sensing and offers an opportunity to understand the neural implementation of sensorimotor integration and control. Little attention has been given to the effects of noise in this system, and we address here limits to the detection of object touch set by noise. In the rodent trigeminal ganglion two types of neurons are considered crucial for whisker-based sensing: 1. Whisking cells, which report the rostrocaudal position of the follicle during whisking motion and independently of object contact, and 2. Touch cells, which report contact with external objects using a binary signal [1]. Temporal combination of Whisking and Touch cell responses allows the animal to infer the rostrocaudal position of external objects with a precision of ~1° [2,3]. Previous work suggested that touch cell responses arise from direct measurements of whisker deflection within the follicle during object contact, by mechanoreceptors in the follicle [3]. The whisker is thought of as a beam constrained by two hinge joints, one at the base of the follicle and one at the exit point, or skin level, of the follicle (See Figure 1). Deflection of the whisker during object contact would thus result in an opposite deflection within the follicle, and which would have to be detected by mechanoreceptors, which could directly drive touch cells.

However, the above biomechanical reasoning for object detection ignores any noise limits to the detectability of beam deformations. To estimate the size of bending that would have to be determined by such mechanoreceptors we analyzed whisker bending using the Euler-Bernoulli beam equation, with relevant parameters taken from the anatomical literature, in order to test this assumption. For a whisker deflection of ~2°, which is factor two above the animal's discrimination threshold, we find a maximum deflection within the follicle on the order of ~1μm. This is likely an overestimate of the true deflection, as we assume a rigid geometry of the follicle and the hinge points. Given the small value of this deflection, and also considering common levels of noise in neurons and detection thresholds in the most sensitive known mechanoreceptors, inner hair cells [4], it is unlikely that object contact is directly detected.

We propose that object contact may be reliably inferred using recursive state estimation of whisker position, akin to findings in human sensorimotor integration [5], by combining sensory information with motor neuron commands moving the follicles. However, during object contact, the force from the object on the whisker would render the internal, forward model biased. The recursive position estimate would thus deviate systematically from a direct position estimate of the whisker orientation from the whisking cells, indicating object contact. The use of recursive state estimation by touch cells would also have additional advantages, such as improving the system performance in the face of noisy sensors and muscle contractions, as well as sensory delays. We predict that receptors driving touch and whisking cells, the putative computations may be carried out monosynaptically using pre-synaptic inhibition and dendritic computation.
